# The circulating IL‐35^+^ regulatory B cells are associated with thyroid associated opthalmopathy

**DOI:** 10.1002/iid3.1304

**Published:** 2024-05-28

**Authors:** Qian Li, Cuixia Yang, Cheng Liu, Yuehui Zhang, Ningyu An, Xiumei Ma, Yang Zheng, Xiaomin Cui, Qian Li

**Affiliations:** ^1^ Department of Ophthalmology, People's Hospital of Ningxia Hui Autonomous Region The Third Affiliated Clinical College of Ningxia Medical University Yinchuan Ningxia Hui Autonomous Region China; ^2^ Medical Science Research Institution of Ningxia Hui Autonomous Region Medical Sci‐Tech Research Center of Ningxia Medical University Yinchuan Ningxia Hui Autonomous Region China

**Keywords:** IL‐35, regulatory B cells, thyroid associated ophthalmology

## Abstract

**Background:**

Thyroid‐associated ophthalmopathy (TAO) is the most common orbital disease in adults, potentially leading to disfigurement and visual impairment. However, the causes of TAO are not fully understood. IL‐35^+^B cells are a newly identified regulatory B cells (Bregs) in maintaining immune balance in various autoimmune diseases. Yet, the influence of IL‐35^+^Bregs in TAO remains unexplored.

**Methods:**

This study enrolled 36 healthy individuals and 14 TAO patients. We isolated peripheral blood mononuclear cells and stimulated them with IL‐35 and CpG for 48 h. Flow cytometry was used to measure the percentages of IL‐35^+^Bregs.

**Results:**

The percentage of circulating IL‐35^+^Bregs was higher in TAO patients, and this increase correlated positively with disease activity. IL‐35 significantly increased the generation of IL‐35^+^Bregs in healthy individuals. However, B cells from TAO patients exhibited potential impairment in transitioning into IL‐35^+^Breg phenotype under IL‐35 stimulation.

**Conclusions:**

Our results suggest a potential role of IL‐35^+^Bregs in the development of TAO, opening new avenues for understanding disease mechanisms and developing therapeutic approaches.

## Graphical abstract

The percentage of circulating IL‐35+Bregs was elevated in thyroid‐associated ophthalmopathy (TAO), and the increase was positively correlated with disease activity. IL‐35 significantly prompted the induction of IL‐35+Bregs in healthy controls, but the B cells in TAO showed potential impairment in the transition of B cells towards IL‐35+Breg phenotype under IL‐35 stimulation.

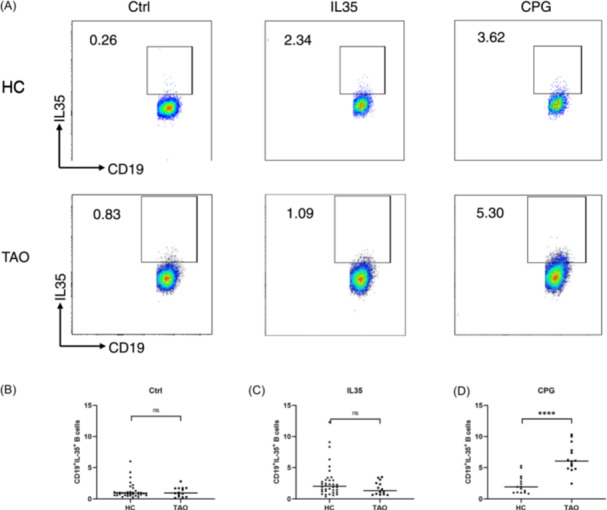

## 1 INTRODUCTION

Thyroid‐associated ophthalmopathy (TAO) is the most common autoimmune orbital disease in adults, often associated with Graves' disease (GD).^1^ The disease progression involves an active phase marked by orbital inflammation and an inactive phase characterized by remodeling of orbital tissues.^2^ This process, though self‐limiting, can lead to disfiguring symptoms such as proptosis, diplopia, strabismus, and even compressive neuropathy,^3^ imposing significant financial and psychosocial burdens on patients.^4^ Consequently, accurate assessment of disease activity and severity is crucial for treatment decision‐making in TAO.

The exact cause of TAO remains incompletely understood. It is widely believed to stem from an autoimmune response in the orbit.^5^ Key components in this process include antibodies against the thyroid‐stimulating hormone receptor (TSHR) and infiltrating T and B cells.^6^ These antibodies bind to TSHR or insulin‐like growth factor 1 receptor (IGF‐1R) on orbital fibroblasts, triggering the release of inflammatory cytokines and recruitment of lymphocytes to the orbit. The cytokines further stimulate the overproduction and deposition of highly hydrophilic glycosaminoglycans and hyaluronic acid in extraocular muscles and orbital fat. Consequently, orbital soft tissues expand, leading to venous reflux obstruction, perpetuating a vicious cycle.^7^ Various drugs targeting biomarkers, such as rituximab for depleting pathogenic CD20^+^B cells,^8^, ^9^ tocilizumab for IL‐6 receptor inhibition, and teprotumumab for IGF‐1R inhibition, have been used in TAO treatment. However, current interventions appear insufficient to halt or reverse the pathological progression of TAO. Hence, there is an urgent need to identify new biomarkers for predicting therapeutic efficacy and establishing better links between pathogenesis and treatment strategies.^10^

The role of regulatory B cells (Bregs) in suppressing immune responses has been demonstrated in various conditions such as experimental autoimmune encephalomyelitis (EAE),^11^ allergic diseases,^12^ transplantation,^13^ and infectious diseases.^14^ In patients with active TAO, the frequency of IL‐10^+^Bregs has been found to increase, while their ability to inhibit IFN‐γ^+^ and IL‐17^+^ T cells in vitro was impaired.^15^ Furthermore, IL‐10^+^Bregs were notably reduced in patients with GD, potentially leading to enhanced activation of Th2 cells and excessive production of thyroid‐stimulating hormone receptor antibodies.^16^

IL‐35^+^Bregs represent a newly identified subset of Bregs that exert their effects by producing IL‐35. Studies in mice lacking either p35 or Epstein–Barr virus‐induced gene 3 (EBI3), key components of IL‐35, demonstrated exacerbation of EAE due to the absence of IL‐35^+^Bregs.^17^ Moreover, patients with chronic hepatitis B virus infection exhibited an increase in the frequency of IL‐35^+^B cells in peripheral blood, which were implicated in dysregulating T cell function through IL‐35‐mediated mechanisms and direct cell‐to‐cell contact.^18^ IL‐35, a member of the IL‐12 family, comprises p35 and EBI3 subunits.^19^ While IL‐12, IL‐23, and IL‐39 are considered pro‐inflammatory members, IL‐27 and IL‐35 are regarded as immune suppressive.^20^ IL‐35 exerts its anti‐inflammatory effects by inhibiting the proliferation of CD4^+^ effector T cells, promoting the differentiation of CD4^+^ effector T cells into regulatory T cells, and stimulating the proliferation of regulatory T cells.^21^, ^22^ Additionally, IL‐35 has been shown to induce the expansion of IL‐10^+^Bregs and IL‐35^+^Bregs.^23^ However, the effects of IL‐35 on Bregs in TAO have not been investigated, and the changes in IL‐35^+^Bregs during the progression of TAO require further elucidation.

In this study, we aimed to determine whether IL‐35 could induce the expansion of IL‐35^+^Bregs, assess the percentage of IL‐35^+^Bregs among total peripheral blood B cells in TAO patients, and explore the relationships between the frequency of IL‐35^+^Bregs and several clinical indicators of TAO.

## 2 MATERIALS AND METHODS

### 2.1 Patients and controls

This cross‐sectional study recruited all participants in accordance with the approved protocol by the clinical Ethics Committees of Peoples' Hospital of Ningxia Hui Autonomous Region. The study adhered to the principles outlined in the Declaration of Helsinki and was conducted from December 2021 to June 2023. Written informed consent was obtained from all participants. Patients diagnosed with TAO based on Bartley criteria ^24^ were included. We selected sex and age as the matching factor to make the TAO patients and healthy adults similar or the same in age and sex composition. We enrolled the TAO patients and healthy adults aged 18–80. After specifying the number of TAO patients in the sex and age layer, we then selected healthy controls (HCs) from healthy adults until the sex and age proportion between the two groups was the same. All the enrolled samples were Han people. A total of 37 healthy adults, matched for age, gender, and ethnicity, served as HCs. Participants with other autoimmune diseases, tumors, or infections were excluded. Additionally, individuals who had received treatment with glucocorticoids, nonsteroidal anti‐inflammatory drugs, or other immunosuppressants within the past 6 months were excluded. The activity of TAO was assessed using the clinical activity score (CAS) criteria, with active disease defined by a CAS score >3.^25^ Disease severity was evaluated based on the NOSPECS classification, with patients rated below grade 3 considered mild, and those with a rating grade of 3–6 were considered as moderate‐severe.^26^

### 2.2 Isolation of PBMCs and induction of bregs

PBMCs were isolated using a density gradient centrifugation method. Briefly, peripheral blood samples were transferred to a 15 mL centrifuge tube and diluted with an equal volume of 1× PBS. An equal volume of human peripheral blood lymphocyte separation solution (Ficoll paque, P8900; Solarbio) was then added to the diluted samples. After thorough mixing, the samples were centrifuged at 700 *g* at 4°C for 30 min. The PBMCs located between the 1× PBS and Ficoll layer were carefully transferred to a new centrifuge tube, and 1× PBS was added to wash the cells. The freshly isolated PBMCs were then incubated in RPMI 1640 medium (Life Technologies) supplemented with 10% fetal bovine serum and 1% penicillin‐streptomycin (both from Life Technologies). PBMCs were seeded into 24‐well plates at a density of 1 × 10^6^ cells/mL/well. Each sample was divided into two groups: a control group cultured with phosphate‐buffered saline (PBS), and an experimental group treated with 10 µg/mL CpG (CpG Oligonucleotides, specifically designed to stimulate TLR 9^27^) (Invitrogen) or rIL‐35 (100 ng/mL) (Sino Biological, Inc.). The cells were cultured at 37°C in a humidified atmosphere containing 5% CO_2_ for 48 h. During the final 5 h of stimulation, 50 ng/mL of phorbol 12‐myristate 13‐acetate and 1 µg/mL of ionomycin (both from Sigma‐Aldrich) were added to the cultures, along with Brefeldin A (Biolegend).

### 2.3 Direct and intracellular staining flow cytometric analysis

PBMCs were suspended in flow cytometry staining buffer (BD Pharmingen) at a final concentration of 10^7^ cells/mL. After incubation with Fixable Viability Stain, BD Horizon™ Brilliant Stain Buffer, and Fc‐blocking antibodies (BD Pharmingen), cells were labeled with antibodies specific for surface markers at 4°C for 30 min. Surface staining was performed with anti‐CD19‐PerCP‐Cy™5.5, anti‐CD38‐BV421 (BD Pharmingen), and anti‐CD138‐APC (Biolegend). For intracellular cytokine staining, the cells were washed, fixed, and permeabilized using the BD Cytofix/Cytoperm™ Fixation/Permeabilization Solution Kit, followed by staining with anti‐IL‐35‐PE antibodies (Biolegend). The gating processes for IL‐35^+^B cells are shown in the supplemental figure. Flow cytometric data were analyzed using FlowJo software version 10.0.7.

### 2.4 Statistical analysis

Results are presented as mean ± SEM unless otherwise stated. Statistical analyses were conducted using the Mann–Whitney *U* test or paired *t*‐test. The Mann–Whitney *U* test was used to compare data from two independent samples where the data was non‐normally distributed. The paired *t*‐test use applied to compare the percentage of IL‐35^+^Bregs between TAO patients and HCs. Spearman rank correlation analysis was used to determine the statistical correlation between variables. A *p*‐value less than 0.05 was considered statistically significant. All statistical analyses were performed using SPSS software (version 21.0.0). Graphs were created using GraphPad Prism 5.

## 3 RESULTS

### 3.1 Demographic characteristics and clinical features of TAO patients

A total of 36 HCs and 14 TAO patients were included in this study. The mean (± SD) ages of HCs and TAO patients were 47.39 ± 9.20 years (range, 22–68 years) and 43.86 ± 11.13 years (range, 24–66 years), respectively. Among HCs, 15 (41.67%) were male, while among TAO patients, 7 (46.67%) were male. There was no significant difference in gender distribution (*p* = .93) or age (*p* = .37) at sampling between HCs and TAO patients. Only two TAO patients had no previous history of thyroid diseases. The average duration of TAO was 30.93 ± 59.98 months (range, 1–240 months), and the duration of thyroid dysfunction was 15.90 ± 13.08 months (range, 1–48 months). Among the TAO patients, 10 cases had previously received treatment with thiamazole, with an average dose of 3.44 ± 1.74 mg. Additionally, two cases had received l‐thyroxine, with an average dose of 75.00 ± 25.00 mg. Among the TAO patients, 3 had active disease, and 11 had inactive disease, with a mean CAS of 1.21 ± 1.90 (range, 0–6). All patients were classified as moderate‐severe according to the NOSPECS score, with a mean score of 3.86 ± 0.52 (range, 3–5). The mean level of total triiodothyronine (TT3), free thyroxine (FT4), and thyroid stimulating hormone (TSH) was 3.76 ± 1.99 ng/mL (range, 2.04–10.40 ng/mL), 2.19 ± 2.26 ng/dL (range, 0.52–9.40 ng/dL), and 5.29 ± 11.45 μIU/mL (range, 0.04‐41.97 μIU/mL), respectively. The normal ranges for FT4, TT3, and TSH are 0.89–1.76 ng/dL, 0.6–1.81 ng/mL, and 0.55–4.78 uIU/mL, respectively. Table 1 presents the clinical features of TAO patients and HCs.

**TABLE 1:** Clinical characteristics of TAO patients and HCs.

Parameters	TAO patients (*n* = 14)	HCs (*n* = 36)
Age (years) (mean ± SD) (range)	43.86 ± 13.13 (24–66)	47.39 ± 9.20 (22–68)
Sex (male/female)	7/7	15/21
Previous history of GD		
Yes	13	NA
No	1	NA
TAO duration (months), (mean ± SD) (range)	30.93 ± 59.98 (1–240)	NA
Thyroid dysfunction duration (months), (mean ± SD) (range)	17.8 ± 14.76 (3–50)	NA
Doses of thiamazole (mg) (mean ± SD)	3.44 ± 1.74	NA
Doses of l‐thyroxine (ug) (mean ± SD)	75.00 ± 25.00	NA
Disease activity		
Active (CAS score > 3)	3	NA
Inactive (CAS score ≤ 3)	11	NA
Disease severity		
Mild (NOSPECS score < 3)	0	NA
Moderate‐severe (NOSPECS score ≥ 3)	14	NA
TT3 (ng/mL)	3.76 ± 1.97	NA
FT4 (ng/dL)	2.19 ± 2.26	NA
TSH (uIU/mL)	5.29 ± 11.44	NA

Abbreviations: CAS, clinical activity score; FT4, free thyroxine; HCs, healthy controls; TAO, thyroid‐associated ophthalmopathy; TSH, thyroid stimulating hormone; TT3, triiodothyronine.

### 3.2 IL‐35 induces the production of IL‐35 in CD19^+^B cells

**FIGURE 1:**
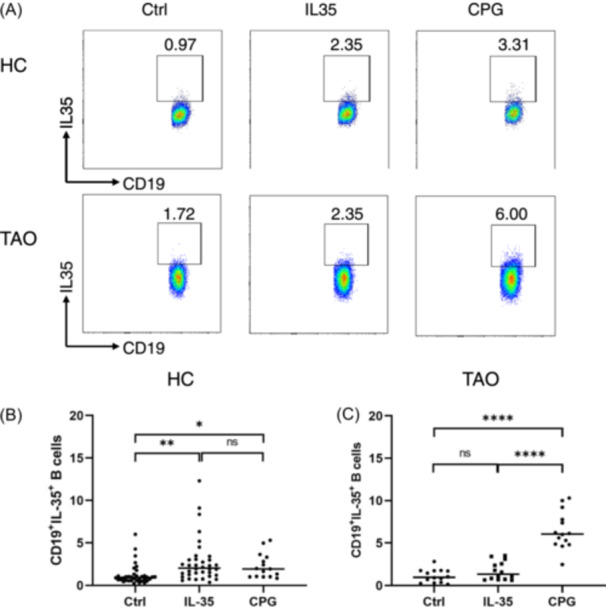
The percentage of IL‐35^+^Bregs induced by IL‐35 and CpG. PBMCs were isolated from 36 healthy individuals and 14 thyroid‐associated ophthalmopathy (TAO) patients. These cells were then stimulated with IL‐35 and CpG for 48 h, with PMA, ionomycin, and BFA added during the final 5 h. (A) Representative dot plots displayed intracellular staining of IL‐35 in CD19^+^ B cells. (B) The percentage of IL‐35^+^B cells after different stimulations was summarized for healthy control (C) and the TAO group. Statistical analysis was performed using an unpaired *t*‐test to determine the significance between different groups. Significance levels were denoted as follows: *, *p* < .05; **, *p* < .01; ****, *p* < .0001; NS, *p* > .05.

To explore the effect of recombinant IL‐35 (rIL‐35) on the expansion of IL‐35‐producing B cells, PBMCs isolated from HCs or TAO patients were cultured either in medium alone or with 100 ng/mL of rIL‐35. Flow cytometry analysis revealed that stimulation with rIL‐35 significantly increased the percentage of IL‐35^+^Bregs from 1.24 ± 1.17 to 2.78 ± 2.55 (*p* < .01) in HCs (Figure 1A,B). In contrast, rIL‐35 stimulation did not lead to a statistically significant increase in the frequency of IL‐35^+^Bregs in TAO patients (Figure 1A,C). These findings suggest that the responsiveness of B cells to rIL‐35 may be impaired in TAO patients.

Previous studies have shown successful induction of B cell differentiation into IL‐10‐producing plasmablasts through CpG stimulation.^28^ Therefore, we further investigated whether CpG induced the expansion of IL‐35^+^Bregs. We observed that activation of PBMCs with CpG resulted in a significantly higher increase in the frequency of IL‐35^+^Bregs in TAO patients. As depicted in Figure 1, CpG stimulation led to a 5.8‐fold increase in the frequency of IL‐35^+^Bregs (from 1.12 ± 0.74 to 6.48 ± 2.15, *p* < .0001). Furthermore, compared to the IL‐35 group, the frequency of IL‐35^+^Bregs also increased by 3.8 times (from 1.71 ± 1.03 to 6.48 ± 2.15, *p* < .0001). However, there was no significant difference in the frequency of IL‐35^+^Bregs between the IL‐35 and CpG groups in HCs (Figure 1A,B). These results suggest that CpG may be a promising candidate for inducing IL‐35^+^Bregs.

### 3.3 Percentage of IL‐35^+^Bregs was increased in TAO patients

**FIGURE 2:**
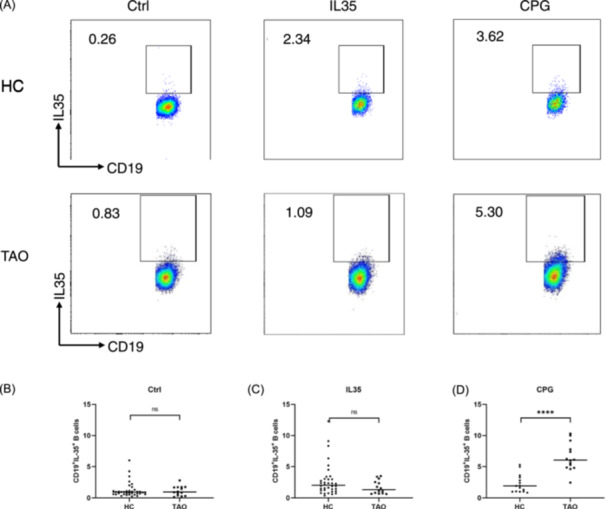
Comparison of the percentage of IL‐35^+^Bregs in healthy control (HC) and thyroid‐associated ophthalmopathy (TAO) group. (A) CD19^+^B cells were gated to determine the proportion of IL‐35+ cells in both the HC and the TAO group. (B) Baseline levels of IL‐35^+^Bregs in both TAO patients and HCs showed no significant difference. (C) The percentage of IL‐35^+^Bregs in HCs was slightly higher than that in TAO patients following induction with IL‐35. (D) The percentage of IL‐35^+^Bregs in TAO patients was significantly higher than that in the HC group following induction with CpG.

We further investigated whether the frequency of IL‐35^+^Bregs differed between TAO patients and HCs. Our findings revealed no significant difference in baseline levels of IL‐35^+^Bregs between TAO patients and HCs (Figure 2B). Although the frequency of IL‐35^+^Bregs in total B cells was slightly higher in HCs (2.78 ± 2.55) compared to TAO patients (1.71 ± 1.03) after stimulation with rIL‐35, this difference was not statistically significant (Figure 2C). Interestingly, following stimulation with CpG, TAO patients exhibited significantly elevated levels of IL‐35^+^Bregs in their peripheral blood compared to HCs (*p* < .001) (Figure 2D).

### 3.4 Positive correlation between the percentage of IL‐35^+^Bregs and disease activity scores in TAO patients

**FIGURE 3:**
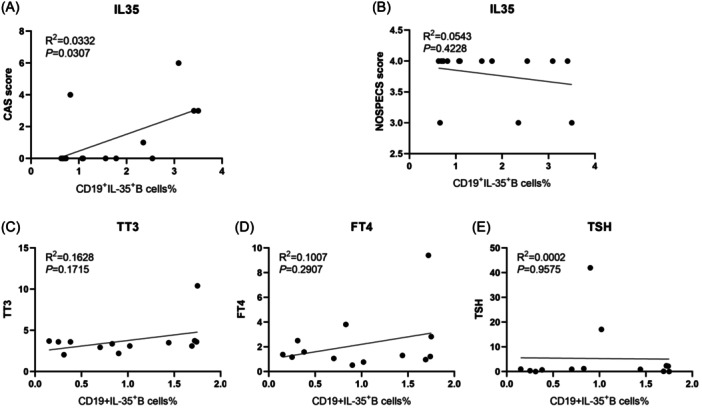
Correlation analyses between the percentage of IL‐35^+^Bregs and clinical indicators. The correlation analyses between the percentage of IL‐35^+^Bregs and (A) thyroid‐associated ophthalmopathy disease activity, (B) disease severity, (C) serum level of TT3, (D) serum level of FT4, and (E) serum level of TSH.

To further understand the significance of IL‐35^+^Bregs in TAO, we examined potential correlations between IL‐35^+^Bregs numbers and TAO disease activity, severity, and thyroid function biomarkers. As shown in Figure 3A, the percentages of IL‐35^+^Bregs were positively correlated with the CAS of TAO patients (*p* = .0307, *r* ^2^ = 0.0332) (*r* ^2^ represented the coefficient of determination in Spearman rank correlation analysis). However, no significant correlations were found between the percentage of IL‐35^+^Bregs and the NOSPECS score (Figure 3B). Similarly, the percentage of IL‐35^+^Bregs showed no correlations with serum levels of TT3, FT4, and TSH (Figure 3C–E). These findings suggest a potential correlation between TAO activity and the concentration of serum IL‐35^+^Bregs.

## 4 DISCUSSION

The sensitivity or resistance to autoimmune diseases hinges on maintaining a balance between pro‐inflammatory responses and regulatory mechanisms of the immune system. While B cells are known for promoting immune responses through antigen presentation, antibody production, and cytokine secretion, they also possess negative regulatory functions by producing immune‐suppressive cytokines such as IL‐10, IL‐35, and TGF‐β.^29^ These anti‐inflammatory B cells are commonly referred to as Bregs. Changes in the proportion of Bregs in peripheral blood have been observed in various types and stages of autoimmune disorders.^30^ However, previous studies have predominantly focused on IL‐10^+^Bregs. For the first time, this study revealed that the proportion of circulating IL‐35^+^Bregs was elevated in TAO patients compared to HCs, which aligns with reports in patients with chronic hepatitis B ^18^ and helminth‐infected patients with multiple sclerosis.^31^ Furthermore, the frequency of IL‐35^+^Bregs increased with the worsening of disease activity in TAO. These findings suggest that IL‐35^+^Bregs may play a role in the pathogenesis of TAO and that their dynamic changes may be associated with TAO inflammation.

Bregs are characterized by their ability to suppress pro‐inflammatory responses. Both immature and mature B cells, as well as plasmablasts, acquire regulatory capacity when exposed to inflammatory signals such as Toll‐like receptor (TLR) ligands and pro‐inflammatory cytokines like IL‐6 and TNF‐α. These signals activate CD40 receptors on the surface of B cells, leading to B cell activation and proliferation.^32^ Studies have shown that B cells from naïve mice can produce significant amounts of IL‐10 upon stimulation with LPS, a TLR4 ligand, or CpG, a TLR9 ligand.^33^ CD19^+^CD25^+^CD71^+^CD73^−^ Bregs have been identified to produce substantial amounts of IL‐10 in response to CpG stimulation, effectively inhibiting the proliferation of antigen‐specific T‐cells in an IL‐10‐dependent manner.^34^ Furthermore, LPS and CpG stimulation can markedly enhance the differentiation of CD25^+^B cells, leading to increased production of IL‐10, IL‐35, and TGF‐β, with a particularly pronounced effect when LPS and CpG are administered together.^35^ In our present study, we also observed a significant increase in circulating IL‐35^+^Bregs following CpG stimulation. Based on these findings and previous studies, it can be inferred that CpG stimulation is essential for the generation of IL‐35^+^Bregs.

In contrast to the specific stimulation of CpG, our study observed a low inducible effect of IL‐35 on IL‐35^+^Bregs in TAO patients. However, in HCs, the frequency of IL‐35^+^Bregs significantly increased after stimulation with IL‐35. IL‐35 triggers the transformation of human B cells into Bregs by activating STAT1 and STAT3 via the IL‐35 receptor, which comprises the IL‐12Rβ2 and IL‐27Rα subunits.^23^ It remains unclear whether IL‐35 has a direct effect on the differentiation of B cells into IL‐35^+^Bregs or if it simply expands a sparse population of IL‐35^+^Bregs. The mechanisms of regulating IL‐35 production by B cells remain poorly understood. Recent studies have suggested that the co‐recruitment of IRF4 and IRF8 transcription factors to immune suppressive genetic loci in B cells may be necessary for differentiation into Bregs that produce IL‐35.^36^ IRF4 and IRF8 are constitutively expressed in B cells and are critical for controlling germinal center events and B cell differentiation.^37^, ^38^ The secretion of IL‐10 by IRF4‐ and IRF8‐deficient Bregs in response to IL‐35 stimulation was found to be limited.^36^ Additionally, another study has shown that IL‐35 can expand the numbers of IL‐35^+^Bregs and IL‐10^+^Bregs, as well as induce IL‐10 production. In our study, we observed a possible hypo‐responsiveness of B cells to IL‐35 in TAO. Thus, based on our findings, we hypothesize that IL‐35 may serve as a pivotal regulatory cytokine in promoting the generation of Bregs by inducing naive B cells to develop into IL‐35^+^ Bregs. However, further efforts are warranted to elucidate the potential molecular mechanism underlying the impaired inductive functions of IL‐35 on B cells in TAO.

The present study has several limitations, including a small sample size and the exclusion of mild TAO patients and GD patients without orbitopathy. In this study, flow cytometry was used to analyze the production of IL‐35 of CD19^+^ B cells. However, potential outliers may occur in the actual experimental operation process, especially when the sample size is small. To reduce the occurrence of this situation, we put strict requirements on the experimental samples. For example, we only use fresh blood samples for further flow cytometry, as the samples that have been cryopreserved may affect the physical state and fluorescence properties of the cells. In addition, we adjusted the cell concentration needs before the experiment, because too low the concentration will directly affect the accuracy and reliability of the detection results. Therefore, when applying the above technique, it is necessary to consider the limitations of the experiment to carry out further experimental verification of the experimental results. Additionally, longitudinal studies assessing the immunosuppressive function of IL‐35^+^Bregs throughout the progression of TAO were not conducted. Furthermore, the inclusion criteria were limited to moderate‐to‐severe TAO patients, restricting the generalizability of the conclusions to an extensive population. Our study solely observed quantitative changes in IL‐35^+^Bregs during TAO pathogenesis, and further investigations are required to elucidate the mechanisms by which IL‐35^+^Bregs contribute to specific autoimmune responses. Consequently, it remains unclear whether these findings illuminate a general mechanism of autoimmune diseases or represent a phenomenon unique to TAO. Ongoing efforts in our center involve larger sample sizes and more comprehensive studies to assess the suppressive function of IL‐35^+^Bregs on inflammatory T cells in vitro.

## 5 CONCLUSION

In conclusion, our research uncovered a significant increase in the percentages of IL‐35^+^Bregs in TAO, along with a positive correlation between elevated IL‐35^+^Bregs levels and disease activity. Furthermore, we observed a reduced inducible potential of peripheral B cells toward IL‐35 in TAO patients compared to HCs. These findings suggest that IL‐35^+^Bregs may play a role in the pathogenesis of TAO.

## AUTHOR CONTRIBUTIONS

**Qian Li**: Conceptualization; data curation; methodology; resources; writing—original draft; writing—review & editing. **Cuixia Yang**: Data curation; supervision; writing—original draft. **Cheng Liu**: Formal analysis; methodology; resources; validation. **Yuehui Zhang**: Data curation; investigation; supervision. **Ningyu An**: Data curation; formal analysis; investigation; resources. **Xiumei Ma**: Data curation; project administration; supervision. **Yang Zheng**: Data curation; supervision. **Xiaomin Cui**: Data curation; investigation. **Qian Li**: Conceptualization; data curation; methodology; resources; writing—original draft; writing—review & editing.

## CONFLICT OF INTEREST STATEMENT

The authors declare no conflict of interest.

## ETHICS STATEMENT

All individuals were recruited according to the protocol that was approved by the clinical Ethics Committees of Peoples' Hospital of Ningxia Hui Autonomous Region. This study adhered to the tenets of the Declaration of Helsinki. Written informed consent was obtained from all participants.

## Supporting information


**Supplemental Figure** Representative flow cytometric profiles illustrating the gating strategy of IL‐35^+^Bregs.

## Data Availability

The data supporting the findings of this study are available upon request to the corresponding authors.
