# Supplementary material for: The circulating IL‐35+ regulatory B cells are associated with thyroid associated opthalmopathy
Source: Immun Inflamm Dis. 2024 May 28;12(5):e1304. doi: 10.1002/iid3.1304 (PMC11131934; doi:10.1002/iid3.1304)
Supplement: Supplementary file 1 — Supplemental Figure Representative flow cytometric profiles illustrating the gating strategy of IL‐35+Bregs. [file IID3-12-e1304-s001.docx]

**Supplemental Figure**

**

CD3

CD19

CD3

CD19
